# The global collaboration on traumatic stress

**DOI:** 10.1080/20008198.2017.1403257

**Published:** 2017-11-30

**Authors:** Ulrich Schnyder, Ingo Schäfer, Helene F. Aakvaag, Dean Ajdukovic, Anne Bakker, Jonathan I. Bisson, Douglas Brewer, Marylène Cloitre, Grete A. Dyb, Paul Frewen, Juliana Lanza, Robyne Le Brocque, Brigitte Lueger-Schuster, Gladys K. Mwiti, Misari Oe, Rita Rosner, Julia Schellong, Jun Shigemura, Kitty Wu, Miranda Olff

**Affiliations:** ^a^ Department of Psychiatry and Psychotherapy, University Hospital Zurich, University of Zurich, Zurich, Switzerland; ^b^ Department of Psychiatry and Psychotherapy, University Medical Center Hamburg-Eppendorf, Hamburg, Germany; ^c^ Norwegian Centre for Violence and Traumatic Stress Studies, Oslo, Norway; ^d^ Department of Psychology, Faculty of Humanities and Social Sciences, University of Zagreb, Zagreb, Croatia; ^e^ Department of Psychiatry, Academic Medical Center at the University of Amsterdam, Amsterdam, The Netherlands; ^f^ Division of Psychological Medicine and Clinical Neurosciences, Cardiff University School of Medicine, Cardiff, UK; ^g^ Ramsay Health Care, The Hollywood Clinic, Perth, Australia; ^h^ National Center for PTSD Dissemination and Training Division, VA Palo Alto Health Care System, Department of Psychiatry and Behavioral Sciences, Stanford University, Menlo Park, CA, USA; ^i^ Institute of Clinical Medicine, Faculty of Medicine, University of Oslo, Oslo, Norway; ^j^ Department of Psychiatry, Western University, London, Canada; ^k^ Argentine Society for Psychotrauma, Buenos Aires, Argentina; ^l^ School of Nursing, Midwifery, and Social Work, University of Queensland, Brisbane, Australia; ^m^ Faculty of Psychology, Clinical Psychology, University of Vienna, Vienna, Austria; ^n^ Oasis Africa Centre for Transformational Psychology & Trauma, Nairobi, Kenya; ^o^ Department of Neuropsychiatry, Kurume University School of Medicine, Kurume, Japan; ^p^ Department of Psychology, Catholic University Eichstätt-Ingolstadt, Eichstätt, Germany; ^q^ Department of Psychotherapy and Psychosomatic Medicine, University Hospital Carl Gustav Carus at the Technische Universität Dresden, Dresden, Germany; ^r^ Department of Psychiatry, National Defense Medical College, Tokorozawa, Japan; ^s^ Asian Society for Traumatic Stress Studies, Hong Kong, China; ^t^ Clinical Psychological Services, Kwai Chung Hospital, Hospital Authority, Hong Kong, China; ^u^ Arq Psychotrauma Expert Group, Diemen, The Netherlands

**Keywords:** Trauma, global collaboration, childhood abuse and neglect, CARTS, refugee mental health, Trauma, Colaboración Global, abuso y negligencia Infantil, CARTS, salud mental para refugiados

## Abstract

Trauma is a global issue. The great majority of the global burden of disease arising from mental health conditions occurs in low- and middle-income countries (LMICs), among populations in political, economic, and/or cultural transition and those struck by forced migration. These mental health problems frequently arise as a result of traumatic events that adversely affect adults, children, and families, including war, mass violence, natural disasters, and accidents. In response to this, the International Society for Traumatic Stress Studies (ISTSS) launched the Global Initiative to have a stronger global impact on trauma-related issues. As part of this initiative, the Global Collaboration was established by representatives of eight professional organizations active in the field of traumatic stress. The group decided to focus on childhood abuse and neglect as its first collaboration. They collected guidelines worldwide, providing the basis for a synthesized core guide for prevention and treatment that can be customized for specific cultural contexts. The resulting ‘Internet information on Childhood Abuse and Neglect’ (iCAN) is a comprehensive guide for adults who have been affected by childhood abuse and neglect, as well as for the survivors’ significant others. It is currently provided in eight languages, and is freely available at the homepage of ISTSS and other websites. A second achievement of the Global Collaboration is the validation of the Computerized Childhood Attachment and Relational Trauma Screen (CARTS), a self-report measure designed to measure occurrences of childhood maltreatment, and its translation into multiple languages, including Croatian, Dutch, French, Georgian, German, Italian, Japanese, Norwegian, Russian, and Spanish. A study is currently planned to collect normative responses to the questionnaire, and to conduct cross-cultural comparisons. The Global Collaboration’s success may be seen as an encouraging step towards a truly global structure in the field of traumatic stress.

## Abstract (es)

El trauma es un problema global. La gran mayoría de la carga mundial de enfermedades derivadas de las enfermedades de salud mental se produce en los países de ingresos medios y bajos (PIMB), entre las poblaciones en transición política, económica y/o cultural y las afectadas por la migración forzada. Estos problemas de salud mental suelen surgir como resultado de acontecimientos traumáticos que afectan de modo adverso a adultos, niños y familias, e incluyen guerras, violencia masiva, desastres naturales y accidentes. En respuesta a esto, la Sociedad Internacional de Estudios de Estrés Traumático (ISTSS) lanzó la Iniciativa Global para tener un impacto global más fuerte en los temas relacionados con el trauma. Como parte de esta iniciativa, la Colaboración Global fue establecida por representantes de ocho organizaciones profesionales activas en el campo del estrés traumático. El grupo decidió centrarse en el abuso y la negligencia infantil como primera colaboración. Recopilaron directrices de todo el mundo, proporcionando la base para un guía central sintetizada de prevención y tratamiento que se puede personalizar para contextos culturales específicos. La ‘información de Internet sobre abuso y abandono infantil’ (iCAN) resultante es una guía completa para adultos que se han visto afectados por abuso y negligencia en la infancia, así como para los seres queridos de los sobrevivientes. Actualmente, se ofrece en ocho idiomas y está disponible gratuitamente en la página de inicio de la ISTSS y otras páginas web. Un segundo logro de la Colaboración Global es la validación de la Detección Computarizada de Apego Infantil y Trauma Relacional (CARTS), una medida de autoinforme diseñada para medir las ocurrencias de maltrato infantil, y su traducción a múltiples idiomas, que incluyen croata, holandés, francés, georgiano, alemán, italiano, japonés, noruego, ruso y español. Actualmente se está planeando un estudio para recopilar respuestas normativas al cuestionario y realizar comparaciones interculturales. El éxito de la Colaboración Global puede verse como un paso estimulante hacia una verdadera estructura global en el campo del estrés traumático.

## 创伤应激的全球合作

创伤是一个全球问题。全球的疾病负担大部分来自于中低收入国家（LMICs）的精神健康问题，集中在处于政治、经济和/或文化过渡期和被迫移民的群体中。创伤事件负面影响着成人、儿童和整个家庭，而这些精神健康问题经常作为这些创伤事件的结果出现。正因为如此，国际创伤应激研究协会（ISTSS）发起了全球倡议，以期对创伤相关问题产生全球化的影响。 作为这项倡议的一部分，全球合作组由八个在创伤应激领域活跃的专业机构组成，决定将第一次合作内容集中在童年虐待和忽视。他们从全世界收集指南，以此为基础提出针对预防和治疗综合的核心指导，并对不同文化内容进行修改。《关于童年虐待和忽视的网络信息（iCAN）》是一个提供给经历过童年虐待和忽视的成人以其重要他人使用的综合性指南。这份指南目前有八种语言版本，可以在ISTSS 的首页和其它网站上免费获得。这次全球合作组的第二项成就是确认了‘童年依恋和关系创伤电脑版筛选工具（CARTS）’ 的有效性。CARTS是一个对是否发生童年虐待的自评工具，已经被翻译成了多种语言：克罗地亚语、荷兰语、法语、格鲁吉亚语、德语、意大利语、日语、挪威语、俄语和西班牙语。目前有一项研究计划收集对这份问卷的常模反应，同时进行跨文化比较。这个全球合作组的成功被看做是在创伤应激领域建立真正的国际结构的推进性鼓励。

## 1. Introduction

Trauma is a global issue (Schnyder, 2013). Traumatic events are common in the lives of people worldwide. When we treat traumatized patients in our own countries, we cannot take for granted that they all speak our language or share our cultural values. Moreover, the great majority of the global burden of disease arising from mental health conditions occurs in low- and middle-income countries (LMICs) (Ferrari et al., 2013; Magruder, Kassam-Adams, Thoresen, & Olff, 2016; Purgato & Olff, 2015), among populations in transition (Hall & Olff, 2016) and those struck by forced migration. These mental health problems frequently arise as a result of traumatic events, including war, mass violence, natural disasters, and accidents. By contrast, only a minority of studies in the field of traumatic stress research are performed in LMICs (Fodor et al., 2014; Schnyder et al., 2016), also reflected in the low number of publications with LMIC authors (Olff & Vermetten, 2013).

In response to this, in 2010, the International Society for Traumatic Stress Studies (ISTSS), as part of its new strategic plan, launched the Global Initiative to have a stronger global impact on trauma-related issues. ISTSS recognized that it could speak with a stronger voice if it represented larger numbers of trauma professionals around the world. ISTSS committed to value worldwide collaboration over competition, and to try to ensure that the needs of all nations are met. One of the action packages approved by the ISTSS Board of Directors was the Global Collaboration. Through this, the ISTSS stimulated a process whereby eight societies in the field of traumatic stress, the Argentina Society for Psychotrauma (SAPsi), the Asian Society for Traumatic Stress Studies (Asian STSS), the Australasian Society for Traumatic Stress Studies (ASTSS), the Canadian Psychological Association Traumatic Stress Section (CPA TSS), the European Society for Traumatic Stress Studies (ESTSS), the German-speaking Society for Psychotraumatology (DeGPT), the ISTSS, and the Japanese Society for Traumatic Stress Studies (JSTSS), agreed to work alongside each other on an equal basis, to identify objectives, facilitate development, and coordinate activities of global importance. Participants felt very strongly that the community of traumatic stress researchers and practitioners should develop collaborations, and ultimately structures, that would enable them to optimally respond to those tasks that are best addressed by means of international collaboration. The Global Collaboration began with the eight societies mentioned above. However, from the very beginning, other groups engaged in traumatic stress related issues were welcomed.

The initial project team, consisting of representatives from the participating societies, first tried to identify various options for a new organizational structure of ISTSS. One model considered was a global collaboration of organizations worldwide with an interest in advancing the field of traumatic stress, with a confederation structure that would include the ISTSS, its current affiliates, plus potentially other associations as well. Another model was the creation of a ‘Global Society for Traumatic Stress Studies’ as a new umbrella organization, in essence a federation similar to how the ESTSS is currently structured. However, not all stakeholders were equally enthusiastic about changing the organizational structure of ISTSS. Therefore, the members of the Global Collaboration considered what they would be able to do better or differently if ISTSS had established a new organizational structure. Keeping in mind the initial purpose of the Global Initiative, and applying the principle of ‘form follows function’, the Global Collaboration decided to convene organizations interested in traumatic stress, and to work alongside each other on an equal basis. Participants would identify objectives, and coordinate activities of global importance. Organizations would be free to determine whether to be involved in particular initiatives.

In November 2012, the Global Collaboration achieved agreement to work collaboratively, focusing on one global issue to start, namely childhood abuse and neglect and the impact of these experiences. This theme was selected by the project team from a long initial list of potential issues of global importance, including motor vehicle accidents, large scale disasters, refugee mental health, and domestic violence. As a result of a face-to-face workshop and a series of conference calls, the project team unanimously agreed that child abuse and neglect are global public health problems that require a global solution. The Global Collaboration decided to collect guidelines worldwide that would provide the basis for a synthesized core guide for prevention and treatment that could be customized for specific cultural contexts. The guide would primarily be aimed at professionals. In addition, the Collaboration would compile an information guide aimed at those affected by childhood abuse and neglect. This was intended to raise global awareness of the issue, and improve the way individuals of all ages who are affected by childhood abuse and neglect are supported, assessed, and treated, leading to significant improvements in health and wellbeing. Capitalizing on the latest developments in technology, the Collaboration aimed to disseminate these guidelines via an application for mobile electronic devices that would facilitate worldwide distribution and cultural customization, or alternatively through the development of an open access website.

This paper is based on a panel discussion that was held at the annual meeting of the 15th conference of the European Society for Traumatic Stress Studies (ESTSS) in Odense, Denmark, in June 2017. The panel introduced the Global Collaboration, the process of working together, the selection of the topics, and other issues. Some of the Global Collaboration’s outcomes were presented, in particular the ‘Internet Information on Childhood Abuse and Neglect’ (iCAN) and the study on a ‘Computerized Childhood Attachment and Relational Trauma Scale’ (CARTS) worldwide. These two projects will now be described in greater detail.

## 2. Internet information on Childhood Abuse and Neglect (iCAN)

There is some evidence that adult survivors of childhood abuse and neglect are reluctant to seek professional help. In a systematic review, the most prominent barriers identified were concerns about stigma, shame, and rejection, but also poor mental health literacy, lack of knowledge, and treatment related fears, e.g. concerns about re-experiencing. In contrast, little is known about facilitators which enable affected individuals to seek help (Kantor, Knefel, & Lueger-Schuster, 2017). Recent developments have highlighted the potential of smartphone applications to provide better access to psychoeducation, self-help strategies, and identifying oneself as a person who needs professional support (Olff, 2015). Therefore, the global collaboration decided to create a tool that would provide affected individuals with knowledge that might enable them to seek professional mental health support.

iCAN was created as an e-pamphlet by members of the global collaboration, located on the homepage of the ISTSS. iCAN offers relevant information to adults with a history of childhood abuse and neglect in a concise format. iCAN includes a definition of childhood trauma, with specific information as to what constitutes childhood abuse, physical abuse, sexual abuse, psychological abuse, and neglect. It also describes the effects childhood trauma can have on survivors even in adulthood, especially with regard to emotional health, mental health, and physical health. A further section focuses on ‘getting help’, discussing the issues of physical/external safety and psychological/emotional/internal safety. iCAN also reflects on aspects of disclosure, since only limited help can be provided without disclosure. Self-help strategies include ‘coping day by day’, addressing issues such as sleep, eating habits, healthy daily structure, self-care, social networks, mindfulness practice, falling back into bad feelings, keeping a diary/journal, and/or expressive writing. The final section is about engaging help from others in terms of peer support and professional help. Overall, iCAN provides comprehensive, scientifically grounded information for adults affected by childhood abuse and neglect. All members of the Global Collaboration approved the content of iCAN before it was translated into various languages. Currently, iCAN is provided in English, Dutch, German, Croatian, Norwegian, Spanish, Japanese, and Chinese. The information is available, free of charge, at the homepages of ISTSS (http://www.istss.org/public-resources/public-education-pamphlets/ican.aspx), ESTSS (www.estss.org), and a number of other websites. For instance, a Chinese version can be found at the AsianSTSS homepage (http://www.asianstss.org). Furthermore, plans for an application of iCAN for mobile electronic devices are under way.

## 3. Computerized Childhood Attachment and Relational Trauma Screen (CARTS)

The CARTS (Frewen, Brown, De Pierro, D’Andrea, & Schore, 2015; Frewen et al., 2013) is a self-report instrument designed to measure occurrences of childhood maltreatment (i.e. physical and emotional abuse of self or other family members, sexual abuse towards the respondent, and ‘bad things’ possibly occurring), in addition to the warmth, security, and supportiveness of the respondents’ family, peers, and other caregivers. A number of face-valid subscales have exhibited good psychometric characteristics and convergent validity with related questionnaires, described previously (Frewen et al., 2013, 2015). An advantage of the questionnaire format of the CARTS, termed a ‘relationally-contextualized approach’ to childhood trauma assessment, is that CARTS items measure not only *what* occurred (e.g. whether the respondent witnessed violence), but additionally to and by *whom* (e.g. *who* was violent to *whom*). Administration of the CARTS is fully automated by an internet website.

A limitation of prior studies using the CARTS, however, is that they have been comprised entirely by English-speaking samples (Frewen et al., 2013, 2015) whereas, as has been discussed above, childhood abuse and neglect are global issues. Accordingly, the ISTSS Global Collaboration has sought to both validate and translate the CARTS into multiple languages, including to date: Croatian, Dutch, French, Georgian, German, Italian, Japanese, Norwegian, Russian, and Spanish. A study is planned to collect normative responses to the questionnaire, and to obtain responses from clinic-referred participants, to further document the prevalence and impact of childhood trauma internationally, as well as to conduct cross cultural comparisons. Indeed, early results from a study comparing responses to the CARTS between Italian and Canadian university students showed that Italian students rated their mothers and fathers as less abusive, but also simultaneously as a poorer source of secure attachment (Simonelli, Sacchi, Cantoni, Brown, & Frewen, 2017). These results suggest that cultural variations can indeed be identified in response to the CARTS attachment- and abuse-scales, suggesting a larger multinational study to better identify the relational context of childhood abuse globally is warranted.

## 4. Conclusions

The aim of this article is to describe and highlight some aspects of the Global Collaboration that was initiated by ISTSS in 2010 and developed into a successful cooperation of – at present – eight professional membership societies in the field of traumatic stress. The consequences of psychological trauma are a global public health challenge. Addressing them requires a joint effort of researchers and clinicians from within the field of traumatic stress as well as a number of experts from other professions and disciplines such as governmental and non-governmental organizations and policy makers. Various strategies to facilitate such a collaborative effort have been discussed in the past. The Global Collaboration as a cooperative of international trauma societies participating on an equal basis has proven successful and has allowed realization of initial projects in the area of childhood abuse, one of the most prominent challenges to global mental health. The Global Collaboration is, therefore, an encouraging step towards the development of a truly global organizational structure in the field of traumatic stress; time will show whether such a new structure, based on equal collaboration, will emerge with form following function.

For future collaboration, apart from translating iCAN into additional languages, a joint research project is currently being considered, looking into child abuse and neglect and adult mental health outcomes from a global perspective. With growing global concerns on issues like child trafficking, migration and war crimes, the Africa orphan generation, breakdown of community support, increase of stress in the family, growing number of single-parent families, internet crime, and so on, child abuse and neglect clearly remain a global shared concern. A coordinated survey could gradually build global understanding and networking so that we can continue to learn from one another while appreciating and respecting our diversities.

Another future topic for the Global Collaboration may be refugee mental health. Like child abuse and neglect, forced migration is a global problem, with an ever-increasing number of people moving across international borders (over 22.5 million in 2016; UNHCR, 2017). A coordinated response, provided by the Global Collaboration, could facilitate continuity of mental health and psychosocial care across borders by setting international standards. This would hopefully improve timely and correct identification of individuals with mental health issues and facilitate their access to evidence based treatments as needed, thus enhancing their capacity to productively integrate into host societies with fewer tensions.

As highlighted above, the Global Collaboration is by no means a closed circle: other organizations in the field of traumatic stress studies are welcome to join in and get involved. Should you be interested, please contact Miranda Olff (m.olff@amc.uva.nl), the current chair of the Global Collaboration.
